# Glucose metabolism and glycosylation link the gut microbiota to autoimmune diseases

**DOI:** 10.3389/fimmu.2022.952398

**Published:** 2022-09-20

**Authors:** Lu Wang, Haojie Xu, Huaxia Yang, Jiaxin Zhou, Lidan Zhao, Fengchun Zhang

**Affiliations:** ^1^ Department of Rheumatology and Clinical Immunology, Peking Union Medical College Hospital, National Clinical Research Center for Dermatologic and Immunologic Diseases (NCRC-DID), Chinese Academy of Medical Sciences, Peking Union Medical College, Beijing, China; ^2^ Key Laboratory of Rheumatology and Clinical Rheumatology, Ministry of Education, Beijing, China; ^3^ State Key Laboratory of Complex Severe and Rare Diseases, Peking Union Medical College Hospital, Beijing, China

**Keywords:** autoimmune diseases, glucose metabolism, glycosylation, gut microbiota, systemic lupus erythematosus

## Abstract

Carbohydrates serve as important energy sources and structural substances for human body as well as for gut microbes. As evidenced by the advances in immunometabolism, glucose metabolism and adenosine triphosphate (ATP) generation are deeply involved in immune cell activation, proliferation, and signaling transduction as well as trafficking and effector functions, thus contributing to immune response programming and assisting in host adaption to microenvironment changes. Increased glucose uptake, aberrant expression of glucose transporter 1 (*e*.*g*., GLU1), and abnormal glycosylation patterns have been identified in autoimmunity and are suggested as partially responsible for the dysregulated immune response and the modification of gut microbiome composition in the autoimmune pathogenesis. The interaction between gut microbiota and host carbohydrate metabolism is complex and bidirectional. Their impact on host immune homeostasis and the development of autoimmune diseases remains to be elucidated. This review summarized the current knowledge on the crosstalk of glucose metabolism and glycosylation in the host with intestinal microbiota and discussed their possible role in the development and progression of autoimmune diseases. Potential therapeutic strategies targeting glucose metabolism and glycosylation in modulating gut ecosystem and treating autoimmune diseases were discussed as well.

## Introduction

Accumulated evidence showed an increased glucose uptake in autoimmune diseases as revealed by ^18^F-FDG PET-CT (1) and implied the impact of distinct glucose metabolic pathways on the immune system. Glucose, serving as an important energy resource and the major substrate for adenosine triphosphate (ATP) generation, is deeply involved in multiple physiological and pathological bioactivities such as immune cell activation, proliferation, signaling transduction, leukocyte trafficking, and cytokine production. Based on the current evidence, immune cells can modulate their cytology to accommodate environmental glucose levels. T cells separated from mice with hyperglycemia showed a more active response to T cell receptor (TCR) stimulation, with accelerated proliferation of CD4^+^ T cells and a higher expression of Th1, Th2, and Th17 cytokines compared with those separated from healthy controls (2), indicating the proinflammatory effects of hyperglycemia circumstances. Glucose deprivation or restricted energy availability steers the metabolic preference from glycolysis towards fatty acid oxidation and consequently adjusts cell fate and polarization; thus, it can be taken as an immune-metabolic checkpoint (3).

During activation, T cells undergo increased glycolysis and oxidative phosphorylation (OXPHOS) to meet the energy demand for proliferation and effector functions (4). Not surprisingly, the upregulation of glycolysis leads to the restricted but rapid generation of ATP and nucleotides, amino acids, and fatty acids synthesis, which are important for immune cells to fulfill multifold tasks, *e*.*g*., cytokine production and proliferation, and switch to inflammatory phenotypes. Increased glucose uptake and accelerated glycolysis are indispensable for immune cells to adapt to microenvironment changes and to cope with external stimuli like infectious pathogens.

Glucose can chemically attach to proteins and nucleic acids without the aid of enzymes, and excessive nonenzymatic glycosylation is involved in multiple biological effects such as inhibiting regulatory molecule binding, decreasing proteolysis susceptibility, influencing nucleic acid function, altering macromolecular endocytosis, and increasing immunogenicity (5). What is more, glycoproteins count on carbohydrates to achieve a proper three-dimensional conformation which is critical for the function and bioactivity of glycoprotein.

Fucosylation is one of the major glycosylation patterns in the human body and is of utmost importance in immune cell maturation, inflammatory cytokine secretion, and gut bacteria adhesion (6). Increased levels of fucosylation had been shown in patients with systemic lupus erythematosus (SLE) and was positively correlated with disease severity (7). Meanwhile, studies have shown that the state of gut microbes is largely regulated by their own carbohydrate modification and host dietary intake as well as host glycosylation (8–16). Both glucose metabolism and microbiota composition as well as the interaction between them are suggested to participate in host immune regulation (17–23). Dysbiosis of intestinal microbiota, such as a lower Shannon diversity index, symbiont translocation, and pathobiont enrichment, has been documented in many types of autoimmune diseases like SLE, rheumatoid arthritis (RA), type 1 diabetes mellitus (T1DM), and multiple sclerosis (MS) with the potential of promoting aberrant immune activation and self-tolerance breakdown (24–29). As a beneficial paradigm, *Akkermansia muciniphila*, a species of human commensal bacterium with mucin-degrading capability that abundantly resides in the mucus layer, can counteract inflammation-associated insulin resistance, display favorable metabolic effects on host glucose and adipose metabolism, reinforce intestinal barrier function, and influence host immune responses. Indeed *A. muciniphila* has shown therapeutic potentials in several inflammation-related diseases, including inflammatory bowel disease (IBD) and diabetes (30–32). In autoimmune diseases characterized by the overactivation of immune cells and aberrant self-attack (33), how glycosylation links glucose metabolism to the multiple physiopathological changes in the immune system and gut ecosystem is of great interest. However, studies on the molecular mechanism linkage of autoimmune diseases with glucose metabolism and glycosylation and their interaction with the gut microbiota were limited.

By analyzing the contribution of distinct glucose metabolic pathways to cell fate determination (34, 35), glycomics provides novel insights into the molecular metabolic pathogenesis of autoimmune diseases. Further research dissecting the complex mutual influence of host glucose metabolism and gut microbiota, together with host immune system programming, might help to exploit new targets or novel intervention strategies to control these diseases. In this review, the interaction of carbohydrate metabolism pathways and glycosylation with the intestinal microbiota and host immune response was discussed based on current knowledge and research. We also discussed their role in the development of autoimmune diseases and the potentials of measures targeting glucose metabolism and microbiota in the treatment of autoimmune diseases.

## Glucose metabolism and glycosylation

### Glucose metabolism

Glucose metabolism is fine-tuned to accommodate the cell demand for energy and biosynthesis. Glycolysis is a fast way to acquire energy, though not as efficient as OXPHOS in the mitochondria, and it is of preference for cells in rapid growth and proliferation. Interestingly, pro-inflammatory cells (*e*.*g*., effector T cells, type 1 macrophage) seemed to more preferentially rely on glycolysis process to acquire ATP for their fate determination and cytokine production, whereas regulatory cells (*e*.*g*., regulatory T cell, type 2 macrophage) might rely more on mitochondrial OXPHOS for their function (36). During glycolysis, glucose is catabolized into pyruvate and lactate which can subsequently be converted into acetyl-CoA to fuel the tricarboxylic acid cycle.

There are three glucose catabolic pathways: aerobic oxidation, anaerobic glycolysis, and pentose phosphate pathway (37). Among all three pathways, hexokinase (HK) is in the first step and the vital enzyme crucial in modulating the intracellular glucose concentration.

As known, mammalian target of rapamycin (mTOR) is also a key regulator in immunometabolism that can regulate glucose uptake and glycolysis in immune cells and, coupled with phosphoinositide 3-kinase/Akt, can modulate diverse cell activities.

Gluconeogenesis typically occurs in the liver but is unveiled recently as a novel function in the gut metabolism. The human intestinal mucosa can express glucose 6-phosphatase and phosphoenolpyruvate carboxykinase gene which is responsible for gluconeogenesis. It is suggested that dietary fiber fermentation products—butyrate and propionate—can induce gluconeogenesis gene expression and benefit body energy homeostasis (38).

### Glycoconjugates and glycosylation

The existence of carbohydrates in the human body is presented in several forms: monosaccharides (*e*.*g*., fructose, galactose, sucrose, ribose), complex carbohydrates (*e*.*g*., oligosaccharides or polysaccharides), and glycoconjugates (*e*.*g*., glycoprotein, glycolipid) (39). Sugars from the daily diet are digested to provide energy source through catabolic pathways or produce structural substances (*e*.*g*., fatty acids, FAs) through anabolic pathways (39). Some intermediate products formed in carbohydrate catabolism become raw materials and carbon frames for synthesizing biological macromolecules such as lipids, proteins, and nucleic acids. Small non-carbohydrate substances can also be converted into carbohydrate molecules or covalently polymerized with carbohydrate molecules to form glycoconjugates in the form of glycoproteins, proteoglycans, glycolipids, and glycophosphatidylinositol-linked proteins (40) and take part in diverse human physiological and biochemical processes.

A layer of glycocalyx covers almost every cell surface in human organs, which is a highly hydrated gel-like layer composed of a meshwork of proteoglycans, glycoproteins, glycosaminoglycans, and glycolipids and assembled with proteins embedded in the plasma membrane to form the external cell surface (41). Mounting evidence has revealed the function of endothelial glycocalyx in maintaining vasculature homeostasis by acting as vascular permeability barrier, physical force transmitter, antagonist of adhesion, and oxidative stress shield, *etc.* (41, 42). Under vascular pathological circumstances, the endothelial glycocalyx can be damaged by hypervolemia or hyperglycemia (43). The glycocalyx is involved in gut epithelial mucosal barrier formation as well, which is mainly composed of sulfated mucin glycoproteins produced by goblet cells (44–46). The gut barrier prevents the invasion of enteric pathogens, and its disruption can cause a “leaky gut” and subject the host to microbial invasion and translocation, which has been reported in multiple autoimmune diseases. In addition, the mucin barrier of the gastrointestinal epithelium might be disrupted under certain a pathological state like IBD, which brings the collapse of its barrier function and promotes bacterial adherence (47, 48). The disruption was found related with both host genetic susceptibility and environmental etiologies (49, 50).

The surfaces of immune cells are also equipped with a mixture of glycoconjugates with complex glycosylation, which play a vital role in regulating immune cell maturation and differentiation (51, 52).

By appending monosaccharide residues to saccharides, peptides, and lipids with glycosidic bonds, catalyzed by glycosyltransferase, glycoconjugates are formed, and the glycosylation process can transform molecular conformation and even alter the antigenicity. Human glycans are basically composed of 10 monosaccharides, including L-fucose, D-galactose, GalNAc, N-acetylglucosamine (GlcNAc), *etc.* (53). Glycosylation of protein is an important way of post-translational modification and ubiquitously involved in human physical activities. Based on whether adding a monosaccharide residue to the free-NH_2_ group or to the oxygen atom of the hydroxyl group in a polypeptide chain, protein glycosylation can be categorized into N-glycosylation and O-glycosylation (47, 53, 54). Though not completely understood, the regulation of the glycosylation process and the glycosyltransferase expression are determined by cell types, respond actively to environmental changes, and participate in cell–cell interactions and distinct protein activity regulation (47, 53). Fucosylation is one of the major forms of glycosylation with in-depth studies and known to participate in the formation of ABO blood group antigen determinants, Lewis antigen determinants, and selectin ligands (6). Fucosylation is accomplished with fucosyltransferases, GDP-fucose synthetic enzymes, and GDP-fucose transporter. Studies found that certain genes in the fucosylation process can upregulate the expression of PD-1 on the cell surface, thus subsequently inactivating the T cells and decreasing the alert threshold towards tumor cells (55). Furthermore, fucosylation of some classifications has been found to have a positive correlation with TNF expression (6), contributing to the inflammation.

## Association of glucose metabolism and glycosylation with host immune response

### Glucose metabolism in immune cells

Immune cells undergo metabolic reprogramming upon antigen challenge. The development, proliferation, and cytokine production of cytotoxic and effector T cells (Teff) rely greatly on glucose metabolism with increased glucose transportation *via* glucose transporter 1 (56), accelerating glycolysis and the bioactive intermediates of the glycolytic pathway (57). The switch of oxidative phosphorylation to aerobic glycolysis is regarded as a hallmark of Teff cell activation (58, 59). Intriguingly, Treg shows unique energetic metabolism by relying on glycolysis to support its proliferation and migration whereas relying on OXPHOS (36) to maintain its suppressive capacity. Glycolysis suppression may reprogram T cell differentiation toward Treg and mTOR inhibitor by inhibiting glucose uptake and inducing hypoxia-inducible factor-1α, subsequently leading to the upregulation of FOXP3 expression, which may partially account for the immune tolerance restoring effects of rapamycin. Foxp3, the well-known transcription factor of Treg, functions to suppress glycolysis and enhance oxidative phosphorylation, thus reprogramming Treg cell metabolism to accommodate low glucose and high lactate conditions (57).

Immune cells also respond to the change of glucose level in the environment with metabolic adjustment, which, in turn, alters their own immune function. Long-term carbohydrate and caloric restriction (CR) lowers the inflammation risks but maintains the T cell repertoire (3, 60). Data from several studies suggested that CR led to the reduction of circulating inflammation markers, including lymphocytes, monocytes, and dendritic cells (DCs) (17–19), but increased the retention of pro-inflammatory monocytes and T cells in the bone marrow (20, 21). Jordan et al. (18) used HK inhibitors to block glycolysis in mice and found a similar shrink of a pool of circulating monocytes, which is mediated by the activation of 5-AMP-activated protein kinase, a key cellular energy sensor (18, 61).

Based on the current evidence, glucose deprivation or restricted energy availability steers the metabolic preference from glycolysis towards fatty acid oxidation and consequently transforms cell fate and polarization; thus, it can be taken as an immune-metabolic checkpoint (3).

### Glycosylation in innate immune response

**Figure 1:**
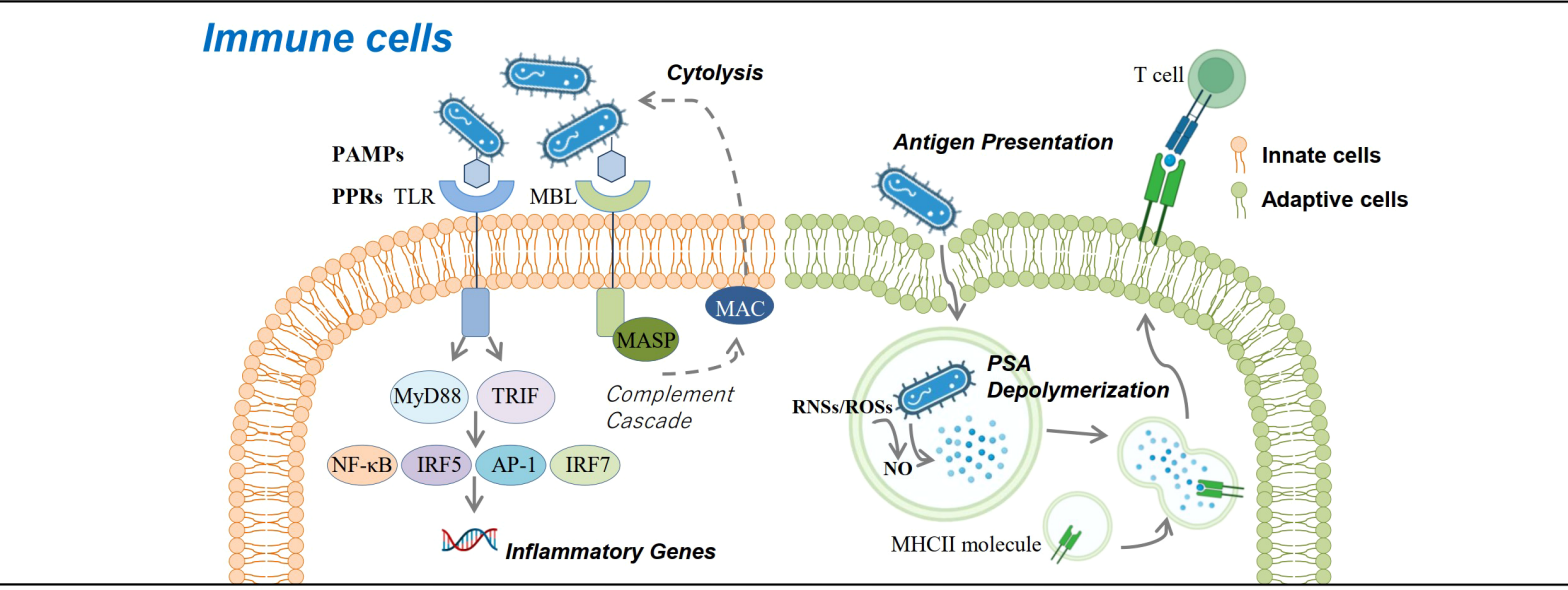
Roles of carbohydrates in immune cells. Carbohydrates are involved in immune response against bacteria at the cellular level. Glycans contained in the glycocalyx of bacteria serve as pathogen-associated molecular patterns that bind with pattern recognition receptors (*e*.*g*., Toll-like receptor and mannose-binding lectin) and activate downstream immune response pathways, either promoting the expression of inflammatory genes or activating the complement cascade and eliminating pathogens. Bacteria can also be presented by antigen-presenting cells *via* the MHCII pathway, which requires NO to help degrade bacterial polysaccharide A into small molecules and present them to CD4+ T cells.

Innate immune response is the first line to defend against microorganism invasion, which is initiated through the recognition of pathogen-associated molecular patterns (PAMPs) or damage-associated molecular patterns (DAMPs) from pathogens with pattern recognition receptors (PRRs) (*e*.*g*., Toll-like receptors, TLRs) either secreted by or anchored in the membrane of innate immune cells (**Table 1**). Most PAMPs have glycan components (47, 62), and bacteria also produce extracellular glycocalyx to mediate cell attachment, protect against antibacterial agents, retain humidity, *etc.* (64)—for example, lipopolysaccharide (LPS) from Gram-negative bacteria, known as endotoxin and the most studied PAMP, is a glycolipid that can induce an acute or chronic inflammatory reaction *via* TLR4 recognition and subsequent NF-κB activation and is associated with a poor prognosis of sepsis. LPS is mainly produced by gut microbes, and increased serum LPS may act as a clue of damaged gut mucus barrier which has been reported in some immune-mediated diseases such as SLE and IBD. Recently, it is suggested that LPS exposure in early life may induce endotoxin tolerance, help suppress Th-2 skewing effects, and modify allergic response, which may act as supportive evidence of the hygiene hypothesis (63). Polysaccharide A (PSA) from *Bacteroides fragilis* is another typical PAMPs, recognized by TLR2, and activates downstream MyD88- and TRIF-dependent pathways, allowing the translocation of transcription factors like NF-κB, AP-1, IRF5, and IRF7 into the nucleus, promoting the expression of inflammatory cytokine genes, and increasing the production of NO and secretion of TNFα in macrophages (22). PRR like TLRs are also heavily glycosylated themselves (62). The glycosylation sites of TLR determine its three-dimensional features and affect its function (47). Lectins are a group of carbohydrate-binding proteins with opsonization capability to mediate the attachment and binding of bacteria by aggregation (23). C-type lectins belonging to PRRs have a specific affinity to different glycosyls and can trigger a series of downstream immunoreactions including T cell activity regulation, cell adhesion, and cytokine secretion (72). Their function as information transmitter in regulating immune response and autoimmunity is coded by diverse glycosylation. According to their affinity to distinct monosaccharide, lectins can be classified into five groups: mannose, galactose/GalNAc, GlcNAc, fucose, and N-acetylneuraminic acid (73). Mannose-binding lectins (MBLs) have a high binding specificity with mannose and GlcNAc. The MBL pathway, as one of the three activation pathways in the complement system, is significant in innate, nonspecific immunity, especially in the initial immune response to sugar-decorated pathogen (74). MBLs and ficolins (another type of lectins in humans that can only bind with GlcNAc) can discriminate self and non-self and bind with carbohydrate groups on the surface of bacteria, fungi, or other pathogens through a COOH terminal carbohydrate recognition domain and activate MBL-associated serine protease (MASP) by a configuration change. MASP1cleaves C3 directly; MASP2 catalyzes the production of C4b2a (C3 convertase), thus triggering the complement cascade by the classical pathway (23). Once C3 is cleaved and C3b is generated, C3b (or C4b, iC3b) is fixed to the bacterial cell surface and binds with complement receptor 1 (CR1) (or CR3, CR4), which is expressed on the surface of phagocytic cells (macrophages, neutrophils, monocytes, *etc.*), and phagocytosis of these bacterial cells is initiated, which is also called opsonization (74–77). The galectins are a group of soluble members of lectin family, excreted by immune cells, endothelial cells, or epithelial cells, and can lead the microbes to the target cells (47, 78) (**Figure 1**).

**Table 1:** Summary about pathogen-associated molecular patterns (PAMPs), damage-associated molecular patterns (DAMPs), and pattern recognition receptors (PRRs) with their characteristics.

	Definition and characteristic	Reference
Pathogen-associated molecular patterns	A common non-specific, highly conserved molecular structure on the surface of pathogens Necessary for the survival and pathogenicity of the pathogen The molecular basis for the recognition of “non-self” by innate immune cells (PRRs)	(22, 47, 62–64)
Damage-associated molecular patterns	The immune system does not distinguish between “self and non-self” but senses danger cells respond to distress (stress, injury, necrosis), danger signals (alarmin), and then respond Including certain proteins or peptides, nucleic acid, unsaturated fatty acids, lipoproteins, purine metabolites, uric acid crystal, cholesterol crystal, and cytokines	(65–68)
Pattern recognition receptors	A class of receptors that directly recognize PAMPs or share specific molecular structures (DAMPs) on the surface of host apoptotic and senesis-damaged cells Present on the surface of various immune cell membranes, intracellular organelles, and serum, such as phagocytes and dendritic cells	(65, 69–71)

DCs are an important component in innate immunity as the professional antigen-presenting cells. Brigitta et al. verified that the nonenzymatic glycosylation of human β2 glycoprotein I (β2GPI) was capable of activating immature DCs, priming of Th2 cell differentiation, and triggering the signaling pathway of ERK, p38 MAPK, and NF-κB. Glycosylation is known to be capable of influencing the immunogenicity of proteins, so it is rational to postulate that glycation of β2GPI may lead to the exposure of cryptic epitope or the formation of neo-determinants to trigger immunogenic DC (79).

Glycolysis induced by mTOR-HIF1α is also involved in neutrophil migration, neutrophil extracellular trap production, and killing activity (80).

### Glycosylation in adaptive immune response

Exploration of the role of carbohydrates and glycosylation on adaptive immune responses revealed that proper MHCII N-glycosylation is critical for the binding and presentation of PSA to CD4+ T cells for their subsequent activation and proliferation (81–83). Mice treated with mannosidase inhibitor to interfere N-glycan complex formation would lose the ability to expand PSA-specific T cells, while the transfer of normally glycosylated APCs to the mice helps restore the ability (84). Other studies found the exposure to PSA results in the upregulation of IFNγ, TNFα, IL-6, CXCL10, and surface and intracellular markers associated with M2 macrophages (85, 86) (**Figure 1**).

**Figure 2:**
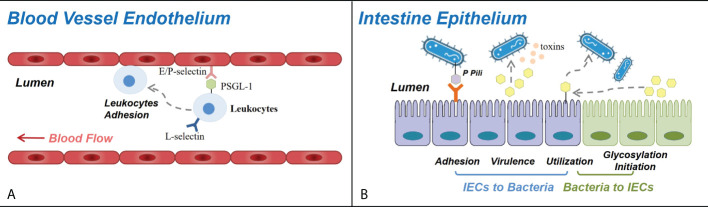
Roles of carbohydrates in blood vessel endothelium and intestine epithelium. **(A)** Selectins and selectin ligands participate in leukocyte trafficking in immune response and autoimmune diseases. E-selectins and P-selectins expressed on the blood vessel wall bind with PSGL-1 and other selectin ligands expressed on the surface of circulating leukocytes, controlling their adhesion and recruitment to the endothelium of CNS venules (in multiple sclerosis), dermal vessels (in autoimmune skin inflammation), ileum and colon vessels (in inflammatory bowel disease), joint synovium (in rheumatoid arthritis), *etc.* **(B)** Carbohydrates and glycosylation mediate the interactions between the bacteria and the intestinal epithelial cells in a variety of approaches. The adhesion and the colonization of bacteria can be mediated by their cell surface glycans binding specifically with receptors on intestinal epithelial cells (IECs). The virulence of bacteria can be influenced as glycogroups on IEC function as bacterial toxin targets, and their breakdown releases products that regulate the expression of bacterial virulence genes. Certain species of bacteria can degrade IEC glycosyls and utilize them, thus influencing the condition of both the bacteria and the intestine epithelium, and several bacterial species can induce the fucosylation initiation of IECs.

During inflammatory response, leukocyte adhesion and extravasation, homing to lymphoid organs, and recruitment to peripheral tissues are closely linked with selectins. Selectins are a group of C-type lectins that are found in the Weibel–Palade bodies of endothelial cells and platelet α-granules (P-selectins), on the surface of skin endothelial cells (E-selectins), and on the surface of most leukocytes (L-selectins) and are inducibly expressed during inflammatory responses (87–90). Most selectin ligands contain a glycan epitope named sialyl Lewis x (sLex) with fucose and sialic acid-decorated carbohydrate chains (90, 91). The main selectin ligand expressed by all calculating leukocytes, P-selectin glycoprotein ligand-1 (PSGL-1), supports the capture and rolling of a broad range of leukocytes (monocytes, DCs, mast cells, NK cells, neutrophils, basophils, eosinophils, activated T cells, and Treg cells) (88, 92). Therefore, malfunction of selectin due to the aberrant structure of selectins and their ligands mediated the impaired leukocyte trafficking which participated in the pathogenesis of autoimmune diseases, including MS, autoimmune skin inflammation, IBD, T1DM, RA, SLE, and systemic sclerosis (88) (**Figure 2A**).

Immune response mediated by antibodies, including phagocytosis, antibody-dependent cell-mediated cytotoxicity (ADCC), and complement activation involves the immunoregulatory role of glycosylation as well (93). IgG is the most abundant immunoglobulin in human serum. Each one of the two heavy chains of an IgG molecule contains an N-linked glycan at Asn-297 in its constant region (Fc), with the ingredients of the glycan including GlcNAc, fucose, mannose, galactose, and sialic acid (94). Distinct glycan modifications affect the shapes of the hinge or the CH_2_–CH_3_ interface of Fc domains and determine the specificity of receptor binding (94, 95). Fc gamma receptor I (FcγRI) is the only type of high-affinity IgG Fc receptors in promoting immune response against bacterial infection and may contribute to several autoimmune diseases. A significant structure to determine its higher affinity is the bond between the receptor D2 domain FG loop and the proximal carbohydrate units (GlcNAc) of Fc glycan from IgG, in the form of a hydrogen bond (95, 96). The FG loop of FcγRII and FcγRIII instead has a distance too far to form a hydrogen bond with the glycan of the IgG Fc domain, which leads to a lower affinity of these two receptors (95). Furthermore, the fucosylated Asn-162 of FcγRIIIA was found to bind with the nonfucosylated glycan of Fc more stably, and studies revealed that FcγRIIIA reached a significantly higher affinity to a fucosylated IgG (absence of core fucose at Asn-297) than fucosylated IgG, which means that the core fucosylation of the Fc domain inhibits the binding of IgG to FcγRIIIA, thus inhibiting ADCC (95, 97–99). On the contrary, the addition of GlcNAc and sialic acid might have the effect of promoting the affinity (100, 101).

## Glucose metabolism and intestinal bacteria

### Host glucose metabolism and glycosylation influence the intestinal ecosystem

Various kinds of carbohydrates and glycosyls exert bidirectional effects on the colonization of different bacteria species in the gut and contribute to the establishment of healthy infant gut microbiota (9, 102, 103). Human intestinal epithelial cells (IEC) mainly carry α (1, 2)-fucosylated glycans, which constitute an important part of the interface between host and microbiota (104). The protective intestinal (also respiratory and genitourinary) mucus layer is built up with large glycoproteins called mucins (MUC) in high density, which is characterized by high O-glycan ratio binding with water (47). MUC2, the major normal mucin in the intestinal tract, forms trimers once secreted and becomes an ideal net-like filter (105). The composition, function, and regulation of mucins are complex and only limitedly understood (45), but mucus glycosylation defection has been implied to be associated with gut flora diversity reduction and composition alteration (8) as exemplified by the finding that Fut2-deficient mice have lower intestinal flora diversity with a shrinking population of an unclassified genus of *Clostridiales* and expanded *Parabacteroides*, *Eubacterium*, *Parasutterella*, *Bacteroides*, and family *Lachnospiraceae* (9, 104), whereas Johansson et al. (106) found that germ-free (GF) mice had increased mucus penetrability and a decreased amount of MUC2 in colon mucus compared to conventional mice but can develop impenetrable mucus and normalized MUC2 after the colonization of conventional cecal flora, demonstrating the contribution of intestinal microbes to the development and glycosylation pattern of mucin.

The carbohydrate modification of mucin domain determines the selection and adhesion of colonized bacteria partially *via* single carbohydrate epitope–protein interactions. As described earlier, bacteria express a layer of extracellular glycocalyx, consisting of adhesive structures named adhesins, whose interaction with host cells are mediated by either protein or glycan (45, 47, 107). The colonization of *Escherichia coli* (*E. coli*) is mediated by its P pili (**Figure 2B**) containing a terminal PapG unit that specifically binds with a digalactoside receptor determinant on gastrointestinal and urinary tract epithelial cells (10, 11, 47). Another study revealed that *E. coli* and *Salmonella typhimurium* bind to glycoprotein 2 on the epithelial M cells *via* bacterial FimH on their pilis (12, 104), and *Vibrio parahaemolyticus* targets the sulfated and fucosylated glycans on the surface of epithelial cells (13, 104).

The virulence of intestinal bacteria is also affected by the glycocalyx structure (**Figure 2B**). *Vibrio cholera* produces cholera toxin which binds to GM1 ganglioside, a type of glycolipid molecule expressed on epithelial cells and which conducts downstream signals to cause watery diarrhea. Galactose and sialic acid compose the sugar moieties of GM1 and might be responsible for the binding with cholera toxin (104, 108). Another study reported that L-fucose released from epithelial cells could inhibit the expression of virulence genes of enterohemorrhagic *E. coli* through a chemical sensing system of the bacteria called FusKR (14, 104).

Carbohydrate fermentation and absorption occurs in the gut, and the decomposition of dietary fibers rely on symbionts residing in the gut (**Figure 2B**). Apart from dietary starch, protein, and fiber (109) in the small intestine, some bacteria species, *e*.*g*., *Bacteroides thetaiotaomicron*, are able to express enzymes to sensor, transport, and degrade glycosyls from the host into short fatty acids (SCFAs) and acquire energy and structure material (45, 110). SCFAs serve as not only energy substrates but also immunomodulators *via* binding to G-protein-coupled receptors (GPCRs) that can induce DC tolerogenic phenotype, promote Treg differentiation, facilitate M2 macrophage conversion, and protect gut homeostasis. Upon stimulation of SCFAs, GPCRs would induce the production of peptide YY, modulating intestinal motility and nutrient absorption (111, 112). Different bacteria have different survival status on O-glycan dense mucus.

In addition, maternal milk contains a thousand types of fucosylated components and a large amount of oligosaccharides (human milk oligosaccharides) which can inhibit the binding of *Campylobacter jejuni* (*C. jejuni*) with fucosyl and prevent diseases associated with it. Both *in vitro* experiment of human epithelial cells and a mouse model infected *in vivo* with *C. jejuni* have proved that 2′-fucosyllactose (2′-FL), the dominant glycosyls in milk, inhibited the colonization, resisted the infection of *C. jejuni*, and mitigated the intestinal mucosa immune response towards *C. jejuni* (113, 114). 2′-FL can also be utilized by other species of bacteria like *Bacteroides* as substrates to produce fucose, lactose, and SCFAs (115).

### Gut microbiota form the glycosylation environment in the intestine

In turn, some intestinal symbiotic bacteria can affect the glucose metabolism and glycosylation status in the host intestinal tract. A germ-free mouse showed defective epithelial α (1, 2)-fucosylation which recovered after commensal bacteria cultivation, implicating that the initiation of epithelial fucosylation partially relies on commensal bacteria (104, 116, 117) (**Figure 2B**). The frequency of fucosylated epithelial cells (F-Ecs) in different locations along the length of the small intestine was found to be positively correlated with the population of microorganisms, and the dependence of fucosylation on bacteria was further verified in both antibiotic-treated mice and GF mice such that a dramatic reduction in F-Ecs frequency and *Fut2* expression was exhibited, which can be restored with antibiotic cessation or conventionalized cultivation (118). Additionally, researchers also found that commensal bacteria selectively induce the fucosylation of columnar epithelial cells and goblet cells instead of Paneth cells (118). Commensal bacteria may have unequilibrim potency in inducing fucosylation, such as *Bacteroides thetaiotaomicron* (*B. thetaiotaomicron*), *Bacteroides fragilis* (*B. fragilis*), *Citrobacter rodentium*, and segmented filamentous bacteria, while *Lactobacillus murinus*, *Bifidobacterium*, and *Peptostreptococcus* cannot initiate fucosylation (104, 117, 119, 120). However, once *Bacteroides* are deficient in fucose metabolic pathway, they lose the function of inducing epithelial fucosylation (104). Apart from commensal bacteria, some pathogenetic bacteria including *S. typhimurium* and *Helicobacter bilis* have also been reported to induce epithelial Fut2 expression and fucosylation (104, 121).

On the flip side, *B. thetaiotaomicron* and *B. fragilis* can also express fucosidase and cut off the terminal and subterminal fucosylation on epithelial cells for self-utilization. This may help to explain why *B. fragilis* has advantages in colonization compared with mutation strains with defects in fucosyl-utilizing enzymes (104, 122).

## The interaction of glucose metabolism and intestinal bacteria contributes to host immune response and autoimmunity

### Intestinal bacteria impact host immune response *via* modifying the glucose metabolism

The human intestinal microbiota is made up of five dominant bacterial phyla: Firmicutes, Bacteroidetes, Actinobacteria, Proteobacteria, and Verrucomicrobia (111). Changes of the intestinal ecosystem have been reported in multiple autoimmune diseases, and the shifted gut microbiota architecture is suggested to be involved in skewing host immune responses through various mechanisms, which have been summarized elsewhere previously (26, 123). **Table 2** shows a summary of the changes in gut micobiota composition in patients with different autoimmune diseases. Specifically, reduced microbial diversity and decreased *Firmicutes/Bacteroides* ratio were found in T1D patients (143), and the gut microbiota composition and the associated metabolites in adult patients with latent autoimmune diabetes (LADA) closely correlated with circulatory autoantibodies, glucose metabolism, islet function, and inflammatory factors (144). PSA from *B. fragilis* can reduce IL-17 expression and upregulate IL-10-producing inducible Tregs. *B. fragilis* colonization was proved to be negatively associated with LPS-induced inflammatory cytokines and chemokines, thus suppressing inflammatory risks (145, 146). An intraperitoneal injection of the bacterial cell wall from certain bacterial species including *Bifidobacterium*, *Streptococcus*, *Lactobacillus*, *etc.*, induced arthritis in susceptible strains of rats, and researchers found that the peptidoglycan component in the bacteria determined their proinflammatory property (147).

**Table 2:** Changes in gut microbiota composition in patients with different autoimmune diseases.

Autoimmune diseases	Increased prevalence	Decreased prevalence	Reference
Rheumatoid arthritis	Eggerthella, Faecalibacterium prausnitzii, Firmicutes, Lactobacillaceae, Bacteroides, Clostridium asparagiforme, Lactobacillus, Holdemania filiformis, Bifidobacterium dentium, Coprobacillus, Eggerthella, Gordonibacter pamelaeae, Ruminococcus lactaris	Bacteroidetes, Bifidobacterium, Clostridium coccoides, Eubacterium, Klebsiella, Haemophilus, Veillonella, Coprococcus, Dialister invisus, Sutterella wadsworthensis, Megamonas hypermegale, Lactobacillus sanfranciscensis	(27, 124–126)
Systemic lupus erythematosus	Eggerthella, Rhodococcus, Klebsiella, Eubacterium, Lachnospiraceae, Genera Rhodococcus, Prevotella, Flavonifractor, ATCC BAA-442, Atopobium rimae, Shuttleworthia satelles, Actinomyces massiliensis, Bacteroides fragilis, Clostridium leptum	Christensenellaceae, Dialister, Odoribacteraceae, Firmicutes, Lactobacillaceaeb, Rikenellaceae, genera Eubacterium, Dialister, Pseudobutyrivibrio	(24, 127–131)
Sjogren syndrome	Bacteroidetes, Firmicutes	Bifidobacterium, Leptotrichia, Escherichia, Shigella, Enterobacter, Fusobacterium	(131–134)
Ankylosing spondylitis	Megamonas, Dorea, Blautia, Clostridiales bacterium, Clostridium bolteae, Clostridium hathewayi, Prevotella copri, Dialister invisus	Lachnospira, Ruminococcus, Clostridium_XlVb, Bifidobacterium adolescentis, Coprococcus, Lachnospiraceae, Roseburia inulinivorans	(135, 136)
Multiple sclerosis	Genus Streptococcus, genera Akkermansia, Clostridium, Blautia, Dorea, Adlercreutzia, genera Oscillibacter, Ruminiclostridium, Anaerostipes, Erysipelatoclostridium, Blautia, Collinsella, Anaerofilum, Flavonifractor, Dorea, Akkermansia, Marvinbryantia	Genus Prevotella, Slackia, genera Bacteroides, Parabacteroides, Butyricimonas, Romboutsia	(137–139)
Type 1 diabetes	Clostridium, Veillonella, Bacteriodetes, Firmicutes, Genera Bacteroides, Veillonella, Alistipes	Lactobacillus, Bifidobacterium, Blautia coccoides/Eubacterium rectale, Prevotella, genera Prevotella, Lactobacillus, Lactococcus, Bifidobacterium, Streptococcus, Akkermansia, Faecalibacterium, Subdoligranulum	(140–142)

Some pathogenetic bacteria in the intestine might stimulate host autoimmune responses *via* molecular mimicry, which may also associate with the glucosyl in bacterial peptide as glucosyl structure and density affect the configuration and even the antigenicity of glycoconjugates. GM1- and GD1a-like LPS on *C. jejuni* surface might be responsible for the generation of anti-GM1b antibodies in Guillain–Barré syndrome patients (148). *Streptococcus agalactiae* can produce sialic acid-capped structures mimicking host glycosylation patterns and bind with sialic acid recognizing Ig-superfamily lectins on DCs, macrophages, and NK cells (51, 149, 150). Group B *Streptococci* can present sialylated glycans on the cell wall as well and prevent attacks from the complement system (150). Studies also proved that the peptides homologous with human RNA binding autoantigen Ro60 and β2-glycoprotein I from *B. thetaiotaomicron* and *Roseburia intestinalis* could provoke autoimmunity and lupus-like manifestations in GF mice models (26, 151).

Goto et al. (118) pointed out that, besides the microbiota–epithelia crosstalk, the stimulation of CD90^+^RORγt^+^ILC3 cells is also essential in the initiation of EC fucosylation. By producing IL-22 and regulating epithelial *Fut2* expression, CD90^+^ILC3 induces epithelial fucosylation and creates a protective barrier against infection in a commensal-bacteria-dependent and commensal-bacteria-independent manner, respectively (118). *Fut2* or α (1, 2)-fucosedeficient mice were found to have an increased risk of *Salmonella typhimurium* infection and vaginal *Candida albicans* (104, 152).

In addition, the mucin barrier of the gastrointestinal epithelium might be disrupted under a certain pathological state like IBD, which brings the collapse of its barrier function and promotes bacterial adherence (35, 36). The disruption was found to be related with both host genetic susceptibility and environmental etiologies (37, 38).

### Disturbed glucose metabolism and glycosylation in autoimmune diseases

There is a broad spectrum of autoimmune diseases but with common features of chronic excessive inflammation, necessitating extra energy and biomaterial supply. Typically, upregulation of GLU1 and increased glucose uptake have been recognized in RA, SLE, and psoriasis. The interference of glycolysis with GLU1 knockout or inhibitor displayed a therapeutic potential and improved the autoimmune phenotype in animal models (153).

In RA patients, CD4^+^ T cells transfer glucose to the pentose phosphate pathway to meet its energy and biosynthesis demand for rapid proliferation (154). In hypoxic synovial cavity, a metabolic shift to glycolysis instead of oxygen-consuming mitochondrial OXPHOS is required for synovium hyperplasia and invasive activity (153). Studies have also found that infiltrating lymphocytes in the synovium of RA mice model expressed increased HK2, the rate-limiting enzyme in glycolysis (155). Using an HK inhibitor, 2-deoxidation-D-glucose (2-DG), can obviously reduce the proportion of Tfh and Th17 cells (156). The galectin-1 expression of RA patients was found to be correlated with the regulation of cell apoptosis, and a galectin-1 injection can help relieve the clinical and histopathological manifestations (157). Another study also revealed the increased galectin-3 levels in the sera and synovial fluids of RA patients (72). From the fucosylation perspective, the expression of Futs catalyzing terminal and subterminal fucosylation was found to be significantly increased in synovial tissues, predominantly in M1 macrophages, which indicated that terminal fucosylation might be a novel hallmark of inflammatory macrophages (158). The antibody glycosylation condition was also altered in RA patients. Parekh et al. (159) found a lower rate of IgG containing galactose residues in RA and OA patients compared with healthy controls.

A similar aberrant glycosylation phenomenon was found in SLE, the prototype of autoimmune disease. Vučković et al. found decreased galactosylation and sialylation of IgG in SLE patients, along with a decreased core fucosylation level and an increased GlcNAc level (160). These alternations lead to a decreased immunosuppressive activity of immunoglobulins and are associated with disease severity (160).

Patients with SLE are prone to present insulin resistance, abnormal glucose tolerance, and diabetes (161), which might be attributed to the metabolic abnormalized effect of inflammatory factors and the functional changes of adipose tissue in SLE patients (162, 163). CD4^+^T cells in lupus patients and lupus-susceptible mice depend on enhanced glycolysis for rapid ATP production to meet their glucose requirements (154). The leukocyte recruitment pathway is mediated by selectin, the level of which is higher in SLE patients’ plasma, indicating that it may contribute to endothelial cell activation and the proinflammatory status in SLE patients. Higher levels of PSGL-1 with enhanced fucosylation on Treg cells in SLE downregulate the TGFβ pathway and inhibit the suppressive activity of Treg cells, which may facilitate self-tolerance breakdown (164).

MS patients, perceived to result from the autoimmune effect of T cell in damaging the myelin sheath, were found to have an elevated level of blood pyruvate in both fasting and postprandial times (165). By treating primary cultures of oligodendrocyte progenitor cells from rat cerebrum with MS-diseased cerebrospinal fluid, Mathur et al. revealed a decreased gene expression related with carbohydrate metabolism, glucose metabolism impairment, and reduced ATP availability for cellular damage repair (166, 167).

### Potential mechanisms underlying the interaction of glucose metabolism and intestinal microbiota changes in autoimmune diseases

Several potential mechanisms have been raised to explain how the interaction between glucose metabolism and intestinal microbiota affects autoimmune diseases. According to the preceding discussion, prior to microbiota colonization, aberrant glucose metabolism and glucosylation condition might occur in patients with autoimmune diseases. On the one hand, this abnormality can cause potential damage to the intestinal mucosa, thus influencing the interaction between gut microbiota and host. On the other hand, aberrant glucose metabolism and glucosylation would also interfere with the function of the host immune system. Genetic factors that participate in the expression of glucose metabolism-related enzymes, like Fut, or intrinsic carbohydrate metabolism disturbance, like mucin disruption in IBD, would lead to the defective formation of the gut protective barrier, which, in turn, alters the gut ecosystem through influences on adhesion, virulence, and metabolism. An abnormal glycosylation level of antibodies either downregulates or upregulates immune responses to certain types of microbes and might damage organs and lead to disease development. *Fut2* might be an important target gene in IBD. By constructing *Fut2*△IEC mice (*Fut2* knockout mice), Tang et al. found that the intestinal epithelium-specific *Fut2* deficiency increases the susceptibility to IBD by regulating the intestinal microbiota and the production of lysolecithin (168). After certain types of microbiota have interacted with the host–microbe interface and colonized, the PAMPs like PSA would be recognized by PRRs and mediate the immune responses. The process of glycosylation might be initiated, thus affecting the intestinal mucosa condition. The microbial metabolism in the gut might produce metabolites such as SCFAs, influencing the immune environment and either improving or exacerbating the disease manifestations (169).

A study revealed that the rapid fucosylation of intestinal epithelium sustained host–commensal symbiosis in sickness. Activation of the TLR signaling pathway leads to the rapid α (1, 2)-fucosylation of mouse IEC. The fucosylated proteins enter the intestinal lumen, and the fucose is then utilized by the intestinal microbiota, which affects bacterial metabolic pathways and decreases the expression of virulence genes (120).

## Prospects of interventions targeting glucose metabolism, glycosylation, and microbiota in autoimmune diseases

Up to now, there are several studies about the treatment of autoimmune diseases targeting different underlying mechanisms, including glucose metabolism, glycosylation, microbiota, *etc.* They might have already shown potential therapeutic effects or indicated future research interests (**Table 3**).

**Table 3:** Potential glucose metabolism, glycosylation, and microbiota targeting therapeutic strategies.

Interventions	Theory	Indication	Reference
Anti-adhesion drugs	Inhibit the interaction between bacteria (*C. jejuni*) and fucosylation Protection of intestinal mucosa against infection	Inflammatory bowel disease	(113)
Fucosylation inhibitors	Competitive inhibitors: block the interaction between fucosyl and selectin Metabolic inhibitors: reduce the process of fucosylation	Sickle cell disease mice model	(6, 170)
Glycoengineering	Glycan modification of IgG Fc domains modulates divergent functions	Arthritis and tumor	(150, 171, 172)
Probiotics, prebiotics, and diet regulation	Probiotics: a group of bacteria that can be beneficial for human immune function, nutrition condition, intestinal microbiota constitution, *etc.*	Rheumatoid arthritis	(173–182)
	Prebiotics: indigestible food ingredients that improve gut microbiota structure by providing carbon sources and metabolic substrates and by adjusting the gut microenvironment	Resisting bacterial infection	
	Diet regulation: a helpful and safe intervention to guide the establishment of healthy gut microbiome	Lupus-prone mice, multiple sclerosis	

### Anti-adhesion drugs

As previously mentioned, the interaction between bacteria (*C. jejuni*) and fucosylation prompts a bacterial infection, and 2′-FL inhibits this adhesion. In a mouse infection model, feeding 2′-FL orally has been proven to lead to a strong protection of the intestinal mucosa against infections (113). This result might indicate the possibility of a novel kind of 2′-FL-derived drug which can be used orally and might be effective in IBD patients.

### Fucosylation inhibitors

Studies have revealed the positive therapeutic effects of small molecules of fucosylation inhibitors. Fucosylation inhibitors can be categorized as competitive inhibitors and metabolic inhibitors; the former blocks the interaction between fucosyl and selectin, while the latter reduces the process of fucosylation (6). Focustatin II, designed as an analog of fucose-1-phosphonate, competitively reduces the fucosylation of therapeutic monoclonal antibodies and improves the efficacy without combination with antibodies (183). The treatment of 2-fluorofocus on a sickle cell disease mice model showed a significant decrease in the adhesion of leukocytes, neutrophils, and sickle red blood cells with the endothelium (170). Other competitive inhibitors include hyperacetylated 6-alkynyl-fucose, hyperacetylated 5-thiofucose (5T-Fuc), *etc.* 5T-Fuc can also play an important role in metabolic inhibition (6).

Glycoregulation therapy with 2-DG and metformin changes the T cell surface glycosylation patterns and enables immune tolerance induction as evidenced by the reduced anti-dsDNA antibody titers and the decreased renal deposition of immune complex as well as the restored tolerance to allografts in lupus-prone mice, probably *via* targeting glycolysis and oxidative phosphorylation (184).

### Glycoengineering

Inspired by the mechanism that glycan modification of IgG Fc domains modulates divergent functions, scientists have come up with the idea of glycoengineering therapy. Pagan et al. increased the sialylation level of IgG *in vivo* in mice models through the administration of engineered glycosyltransferases and found that it successfully exerted an anti-inflammatory effect and was effective at relieving arthritis (171, 172). Furthermore, activated platelets were found to be significant ingredient donors of *in vivo* sialylation, and coordinately giving clopidogrel or other platelet inhibitor drugs would inhibit the anti-inflammatory effect of this glycoengineering therapy though the platelets themselves have pro-inflammatory effects (171, 185, 186). Glycoengineering has also attracted interest in the field of tumor as the mechanism of immune evasion of tumor cells also involves the sialylation of related proteins (150).

### Probiotics, prebiotics, and diet regulation

Probiotics are a group of bacteria that can be beneficial for human immune function, nutritional condition, intestinal microbiota constitution, *etc.* Prebiotics are indigestible food ingredients that improve the gut microbiota structure by providing carbon sources, making metabolic substrates available for the gut microbiota to utilize, and adjusting the gut microenvironment, such as in terms of pH value, gastrointestinal absorption, and enzyme hydrolysis (173, 174). Fructo-oligosaccharide (FOS) is a well-studied effective prebiotic consisting of one glucose and one fructose, and it has shown multiple beneficial effects in several animal models including the following: (a) resisting bacterial infection such as that of enterotoxigenic *E. coli* (176), (b) promoting the colonization of probiotics like *Bifidobacterium* and *Lactobacillus* (177–180), (c) increasing the production of SCFAs by the use of these probiotics (180), and (d) downregulating the pro-inflammatory cytokine expression and protecting the mucosal barrier (181). Besides FOS, inulin, galacto-oligosaccharides, and some foods containing indigestible β-glucans such as termites, a kind of Chinese traditional medicine, also have a similar effect (174).

As has been proved that chemically synthesized oligosaccharides with core fucosylation have the function of promoting *Bifidobacterium* and *Lactobacillus* growth, it is put forward that the core fucosylation in maternal milk is a promising prebiotic for infants (102). Gestational diabetes mellitus during maternity results in higher fucosylation and sialylation levels in milk that affects the immune function of infants, which indicates the importance of guidance in beneficial nutrition and healthcare of gestating women (187).

Furthermore, diet regulation has been raised as a helpful and safe intervention to guide the establishment of a healthy gut microbiome and restore beneficial metabolism. Resistant starch or rich-fiber diet has shown many benefits in lessening the inflammation status and improving metabolic aberrance by retarding glucose absorption, strengthening the gut barrier, flourishing beneficial commensals, and reducing cytokine release. Kearney et al. (188) reported an increase of relative abundance of *Bacteroides plebeius* by more than two orders of magnitude in mice fed with a diet containing 1% raw seaweed. Dietary resistant starch is proven to be beneficial to lupus-prone hosts by suppressing the abundance and translocation of *Lactobacillus reuteri* which drives autoimmunity (182). Studies also report that the Mediterranean-style diet, characterized by rich unsaturated fatty acids and fibers, has anti-inflammatory effects and produces a beneficial effect to relieve the symptoms of MS patients (189, 190).

### Other therapy strategies

Though carbohydrates play a vital role in physiological and pathological events, they can be easily eliminated by the digestive and urinary systems; therefore, the development of drugs directly derived from carbohydrates was limited. Some carbohydrate-derived drugs, like sodium hyaluronate (decreasing the risks of infection and pain in arthroscopic debridement) (191) and sulfated glycosaminoglycans (binding to antithrombin and promoting anticoagulation in thrombosis) (40, 192), have been indicated as beneficial for osteoarthritis. Researchers also attempted to deliver carbohydrates orally or by injection and observe their pharmacokinetics and effects. Pozharitskaya et al. intragastrically administrated fucoidan to rats and found that it accumulated firstly in the kidney, spleen, and liver and had a relatively long elimination time from circulation, thus revealing the possibility of fucoidan-derived drugs (193). Wu et al. (194) treated induced obese mice with fucose by intragastric administration and found decreased body weight gain, fat accumulation, and hepatic triglyceride elevation in mice.

In addition, several novel oral heptadecaglycoside antibiotics have been studied and developed. Saccharomicins A and B, isolated from *Saccharothrix espanaensis*, showed inhibition effects to pathogenetic bacteria growth both *in vivo* and *in vitro* (195). The microscopic observations showed that the treatment of these drugs could inhibit the synthesis of DNA, RNA, and proteins in bacteria (195, 196).

In conclusion, the mutual influence of glucose metabolism, glycosylation, and intestinal microbes is complicated, and their contributions to autoimmune diseases are largely unclarified. A well-defined integrated network can improve our substantial understanding about the dynamic change of immunity and metabolism under the era of microbiota. From the perspective of the synergistic effect of glucose metabolism, glycosylation, and intestinal microbiota, new inspirations and intervention strategy may be developed for autoimmune diseases.

## Author contributions

All authors were involved in drafting this paper or revising it critically for key intellectual content. JZ and LZ had full access to all the data in the article. Conception: LZ and JZ; methodology: LZ, HX, and LW; interpretation and writing: LW, HX, HY, and LZ; and supervision: LZ and JZ. All authors contributed to the article and approved the submitted version.

## Funding

This study was supported by the National Natural Science Foundation of China (82071840) and CAMS Innovation Fund for Medical Sciences (CIFMS) (2020-I2M-C&T-B-013).

## Conflict of interest

The authors declare that the research was conducted in the absence of any commercial or financial relationships that could be construed as a potential conflict of interest.

## Publisher’s note

All claims expressed in this article are solely those of the authors and do not necessarily represent those of their affiliated organizations, or those of the publisher, the editors and the reviewers. Any product that may be evaluated in this article, or claim that may be made by its manufacturer, is not guaranteed or endorsed by the publisher.
